# AI-based multi-PRS models outperform classical single-PRS models

**DOI:** 10.3389/fgene.2023.1217860

**Published:** 2023-06-27

**Authors:** Jan Henric Klau, Carlo Maj, Hannah Klinkhammer, Peter M. Krawitz, Andreas Mayr, Axel M. Hillmer, Johannes Schumacher, Dominik Heider

**Affiliations:** ^1^ Department of Mathematics and Computer Science, University of Marburg, Marburg, Germany; ^2^ Center for Human Genetics, University of Marburg, Marburg, Germany; ^3^ Institute for Genomic Statistics and Bioinformatics, Medical Faculty, University Bonn, Bonn, Germany; ^4^ Institute for Medical Biometry, Informatics and Epidemiology, Medical Faculty, University Bonn, Bonn, Germany; ^5^ Institute of Pathology, Faculty of Medicine, University of Cologne, Cologne, Germany

**Keywords:** polygenic risk score, machine learning, deep learning, breast cancer, regression

## Abstract

Polygenic risk scores (PRS) calculate the risk for a specific disease based on the weighted sum of associated alleles from different genetic loci in the germline estimated by regression models. Recent advances in genetics made it possible to create polygenic predictors of complex human traits, including risks for many important complex diseases, such as cancer, diabetes, or cardiovascular diseases, typically influenced by many genetic variants, each of which has a negligible effect on overall risk. In the current study, we analyzed whether adding additional PRS from other diseases to the prediction models and replacing the regressions with machine learning models can improve overall predictive performance. Results showed that multi-PRS models outperform single-PRS models significantly on different diseases. Moreover, replacing regression models with machine learning models, i.e., deep learning, can also improve overall accuracy.

## 1 Introduction

Disease prevention is a crucial part of medical care. It reduces the costs for the healthcare system and reduces the number of hospitalization and deaths (Kahn et al., 2008). For targeted preventive measures, it is necessary to determine the individual risks for certain diseases. In addition to age, sex, and lifestyle, genetic factors play an important role in determining the individual risk. Polygenic risk scores (PRS) are used to take multivariate genomic information into consideration and can be used for the selection of a targeted treatment in personalized medicine (Lambert et al., 2019; Lewis and Vassos, 2020; Schröder et al., 2022).

PRS are typically modeled as a regression task by calculating a weighted sum of all genotypes and their corresponding estimated effect size. Relevant single nucleotide polymorphisms are discovered by genome-wide association studies (GWAS). For individual risk prediction, another regression model is built based on the previously calculated PRS and other covariates, such as age, sex, and lifestyle (e.g., smoking and alcohol consumption) (Choi et al., 2020).

In recent years, machine learning (ML) has led to numerous advances in medicine (MacEachern and Forkert, 2021) due to the ability to train models on complex problems and being able to handle large amounts of data. These models have been used in various applications, e.g., oncology (Bibault et al., 2016), pathology (Madabhushi and Lee, 2016; Coudray et al., 2018), diabetes (Spänig et al., 2019), human genetics (Libbrecht and Noble, 2015), and infectious diseases (Riemenschneider et al., 2016b; Ren et al., 2021) as part of a growing trend toward personalized/precision medicine.

In this study, we trained multiple models, i.e., ridge regression (RR), random forests (RFs), and deep neural networks (DNNs), to predict an individual’s phenotype for the following diseases: breast cancer (BC), coronary artery disease (CAD), and type 2 diabetes (T2D). We selected those three common chronic diseases to demonstrate the usefulness of our approach for different diseases. For instance, breast cancer is diagnosed in approximately 2.3 million women yearly. Cardiovascular diseases are the leading cause of death globally. Coronary artery disease affects approximately 126 million individuals, with 7.2 million deaths each year. Diabetes affects approximately 425 million people worldwide.

The inclusion of additional PRS has been shown to improve the prediction of traits and diseases (Krapohl et al., 2017) (Sinnott-Armstrong et al., 2021) (Abraham et al., 2019), psychological diseases, such as schizophrenia, bipolar disorder, or depression (Rodriguez et al., 2022), the risk of exposure to bullying (Schoeler et al., 2019), and hazard ratios (Meisner et al., 2020). Thus, we further evaluated the inclusion of 139 additional PRS in a multi-PRS approach to the prediction of the previously mentioned diseases. The additionally used PRS do not have to be directly associated with the investigated disease (Sinnott-Armstrong et al., 2021). Including these PRS, even if the phenotypes appear to be unrelated, may be beneficial as similar underlying biological mechanisms may be involved.

## 2 Materials and methods

**FIGURE 1:**
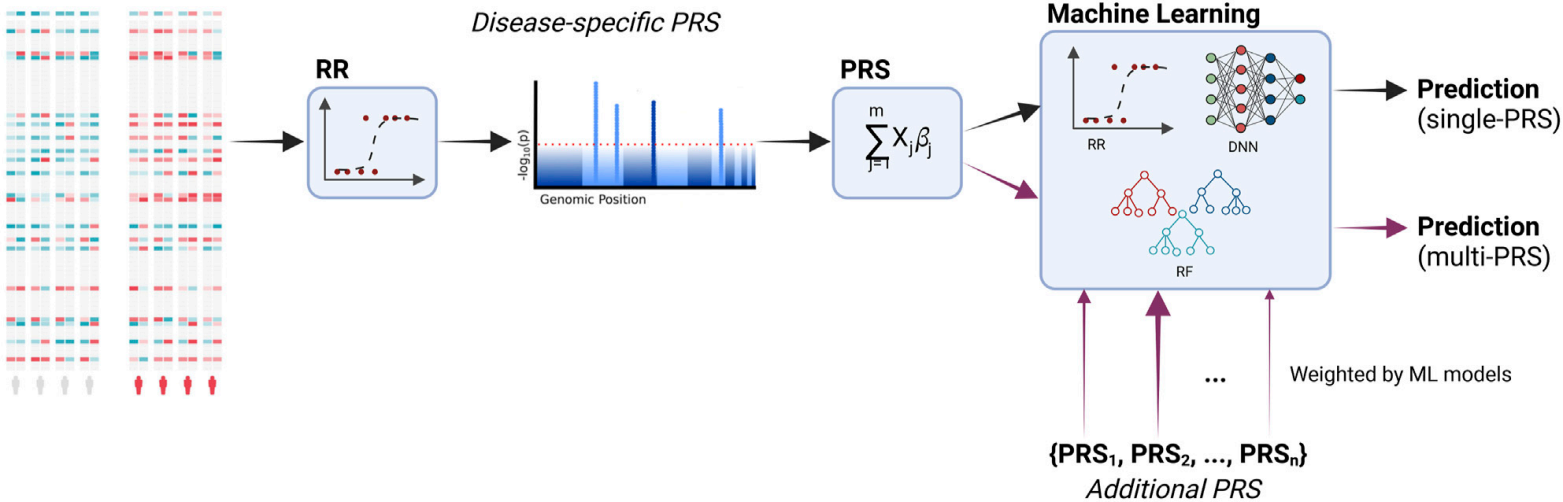
Workflow of the study. PRS are calculated based on the associated genetic loci (i.e., SNPs, single-nucleotide polymorphisms). Significant loci are identified via a regression model. These loci are then used to calculate the PRS based on a linear combination. Additional PRS for other diseases are incorporated into the final predictive model. During training, the models learn to distinguish between relevant and irrelevant features, including the additional PRS. Moreover, we compare the typically used ridge regression with machine learning models, namely, deep neural networks and random forests. Created with BioRender.com.

The workflow of the current study is shown in Figure 1. We incorporated additional PRS into the predictive models and, additionally, compared different machine learning models to the regression models that are typically used in PRS.

### 2.1 Data

This research has been conducted using the UK Biobank resource (Bycroft et al., 2018) under application number 81202. The UK Biobank is a large-scale cohort study covering a huge prospective sample (*n* > 500,000) of the British general population, including both genotype and phenotype (health-related outcomes) data. We used the imputed UK Biobank data which include ˜96 million variants.

We excluded available genotype data outliers for heterozygosity (F within three standard deviations (SD) from the mean), sample genotype missing rates (>2%), and discordant reported sex vs. genotypic sex. Allele frequency MAF < 0.1% was removed. Variants not in the Hardy–Weinberg equilibrium (*p*-value <10^–6^) were excluded.

In total, 139 PRS (Supplementary Table S1) for different phenotypes, e.g., lung cancer (PGS000078), venous thromboembolism (PGS000043), and fasting glucose (PGS000305), were computed using PLINK (Chang et al., 2015) score function, and the corresponding effect alleles and beta coefficients were retrieved from the PGS Catalog (https://www.pgscatalog.org/). The PRS are therefore based on a linear additive combination of effect alleles and are characterized by a normal distribution. Due to the great abundance of SNPs in the imputed UK Biobank, adequate coverage was ensured.

The additional 139 PRS were added as additional input features without any pre-selection to enable a data-driven approach without any subject-matter knowledge. Therefore, we included all PRS that were available in the PGS Catalog at the time we started the project. The underlying idea is that different diseases can share different pathways, e.g., inflammatory pathways, or even comorbidities. Selection of PRS according to phenotype association with the investigated disease, though more interpretable, can potentially miss relevant information. By using multiple risk scores, we were able to capture the interdependencies in a data-driven approach by machine learning models. PRS that were calculated on the same UK Biobank cohort for one of our target diseases could induce overfitting or circularity. For PRS that were calculated on the UK Biobank cohort, but for different diseases, this would only affect the control group. Therefore, these effects are, if at all, of very little impact.

From the phenotypic data, we derived the case/control status for three diseases, namely, BC, CAD, and T2D. BC cases were women based on self-report in an interview with a trained nurse and/or BC-related ICD-9 codes (174 or 174.9) or ICD-10 codes (C50.X) in hospitalization records. CAD cases were individuals with myocardial infarction based on self-report or hospital admission diagnosis according to ICD-9 codes of 410.X, 411.0, 412.X, or 429.79 or ICD-10 codes of I21.X, I22.X, I23.X, I24.1, or I25.2 in hospitalization records and/or with coronary artery bypass grafting (K40.1–40.4, K41.1–41.4, or K45.1–45.5) or coronary angioplasty with or without stenting (K49.1–49.2, K49.8–49.9, K50.2, K75.1–75.4, or K75.8–75.9). T2D cases were samples based on self-report in an interview with a trained nurse or an ICD-10 code of E11.X in hospitalization records. For controls, all individuals without the phenotype were considered (for BC, the analysis was restricted only to women).

In order to limit the confounding due to the genetic background, the analysis was restricted only to individuals with White British origin (Field 21000) and with European genetic ancestry according to the principal components provided by UK Biobank (Field 22006), and among the remaining samples, to account for the residual population stratification, we considered the principal components (PCs) as computed in UK Biobank (Field 22009). The total number of individuals in the data set amounts to 429,466, while the number of patients for the three diseases, BC, CAD, and T2D, are 13,679, 23,033, and 24,241, respectively (Table 1).

**TABLE 1:** Number of individuals in the case and control groups.

	BC (female only)	CAD	T2D
Cases	13,679	23,033	24,241
Controls	232,424	406,433	405,225

### 2.2 Data preparation

We included the following features into the model training: corresponding PRS (i.e., BC-PRS (PGS000015), CAD-PRS (PGS000013), or T2D-PRS (PGS000014), respectively), first 10 PCs, age, sex, and the genotyping array. Categorical features such as sex and genotyping array were one-hot encoded, while all other features were normalized to values between 0 and 1. For the prediction of BC, only female individuals were included, and sex was removed as an input feature. For the multi-PRS approach, 139 additional PRS (e.g., lung cancer (PGS000078), venous thromboembolism (PGS000043), and fasting glucose (PGS000305)) were included in the data set.

### 2.3 Model development

The data sets were split for each individual disease into training and test sets (75:25) using a stratified approach to preserve a disease’s prevalence within each data set. This was repeated three times with different seeds to assert the robustness of the model’s prediction on previously unseen data sets. The training set was then used in a stratified 10-fold nested cross-validation. Due to the class imbalance in the data, the training data set was upsampled within the nested cross-validation (Beinecke and Heider, 2021). We compared multiple methods in our study: RR, RF, and DNN.

#### 2.3.1 Ridge regression

Ridge regression (RR) is a statistical method that includes a penalty parameter, rendering it more stable when input features are correlated compared to other regression models. RR is typically used in calculating PRS. For the RR, we used the scikit-learn library version 0.23.2 (Pedregosa et al., 2011).

#### 2.3.2 Random forests

Random forests (RFs) are proven non-linear classifiers that have been shown to produce good results even in small-*n*-large-*p* scenarios in biomedical classification (Riemenschneider et al., 2016a; Anastasiou et al., 2017). They are based on multiple decision trees that are combined via a majority vote (Breiman, 2001). We used the implementation of the scikit-learn library version 0.23.2 (Pedregosa et al., 2011).

#### 2.3.3 Deep neural networks

Deep neural networks (DNNs) are modeled after biological neurons and consist of multiple layers of artificial neurons. In our study, we used only deep feed-forward networks, where each of these neurons has multiple inputs via weighted connections to previous neurons and calculates an output on the sum of all inputs and with a given activation function. The first layer is called the input layer and is fed with the training features, while the last layer is called the output layer and provides the prediction of the network. These two layers are connected by several so-called hidden layers. All DNNs were implemented using the PyTorch library version 1.7.1 (Paszke et al., 2019).

#### 2.3.4 Hyperparameter optimization

Hyperparameter optimization of all models was carried out within the nested cross-validation. For the DNNs, we evaluated different topologies, ranging from 3 to 6 layers and 2 to 512 neurons per layer. Learning rates of 1 × 10^−5^, 1 × 10^−4^, and 1 × 10^−3^ were tested. The loss function used was BCELoss. RFs were optimized with regard to the number of trees (100, 250, 500, and 1,000) and the maximum depth per tree (default, 10, 25, and 50). For RR models, the number of iterations (default, 100, 500, 1,000, and 5,000) was optimized.

After optimizing the hyperparameters in the 10-fold nested cross-validation, models were trained on the full training set using the optimal hyperparameters and then used to predict the test set. Models were evaluated based on the area under the receiver operating characteristic curve (AUC) and accuracy on the test set averaged over three random seeds.

## 3 Results

For the DNNs, no single best topology for all tasks was found (Table 2). The best learning rate for all DNN models was 1 × 10^−4^. The best topology for the single-PRS approach for all data sets is 16-8-4-1, while the best topology for the multi-PRS approach is 8-4-4-1 for CAD and T2D and 16-8-4-1 for BC. The rectified linear unit (ReLU) was used as an activation function after all layers, except for the output layer, where the sigmoid function was used. The models performed best after 100 epochs of training. The training of single-PRS models took approximately 8 min, while multi-PRS trainings took approximately 10 min, resulting in a total training time of approximately 80 and 100 min, respectively, for a 10-fold cross-validation. Due to the lower amount of samples for BC, training times were halved for these models.

**TABLE 2:** Comparison of DNN, RF, and RR on the three data sets, BC, CAD, and T2D, for single- and multi-PRS approaches. Evaluation based on AUC and accuracy according to Khera et al. (2018). Values are shown as mean ± SD.

Method	Disease	PRS mode	Accuracy	AUC
DNN	BC	Single-PRS	0.613 ± 0.021	0.653 ± 0.004
DNN	BC	Multi-PRS	0.628 ± 0.024	0.668 ± 0.001
RF	BC	Single-PRS	0.592 ± 0.015	0.626 ± 0.005
RF	BC	Multi-PRS	0.609 ± 0.009	0.648 ± 0.002
RR	BC	Single-PRS	0.598 ± 0.007	0.652 ± 0.004
RR	BC	Multi-PRS	0.612 ± 0.011	0.670 ± 0.002
DNN	CAD	Single-PRS	0.694 ± 0.009	0.785 ± 0.002
DNN	CAD	Multi-PRS	0.698 ± 0.012	0.790 ± 0.002
RF	CAD	Single-PRS	0.674 ± 0.002	0.765 ± 0.003
RF	CAD	Multi-PRS	0.683 ± 0.004	0.768 ± 0.002
RR	CAD	Single-PRS	0.696 ± 0.004	0.785 ± 0.002
RR	CAD	Multi-PRS	0.693 ± 0.004	0.790 ± 0.002
DNN	T2D	Single-PRS	0.626 ± 0.017	0.703 ± 0.002
DNN	T2D	Multi-PRS	0.653 ± 0.010	0.716 ± 0.003
RF	T2D	Single-PRS	0.607 ± 0 014	0.675 ± 0.001
RF	T2D	Multi-PRS	0.610 ± 0.001	0.686 ± 0.002
RR	T2D	Single-PRS	0.636 ± 0.007	0.703 ± 0.002
RR	T2D	Multi-PRS	0.636 ± 0.008	0.716 ± 0.002

For the RF models, the best predictions were obtained with 500 trees, while all other parameters were left at the default value. For the RR models, all parameters were left at the default value.

It turned out that the DNNs performed equally well or outperformed RR in all data sets, in particular for the multi-PRS approach. RF did not outperform RR in any data set, neither as single-PRS nor as multi-PRS. In fact, RF performed significantly worse for all data sets and PRS modes with approximately 2% lower AUC and accuracy values than RR and DNNs.

For instance, the DNNs reached an accuracy of 0.653 ± 0.010 compared to 0.636 ± 0.008 for RR for the T2D data set using the multi-PRS approach. For the BC data set, the DNN reached an accuracy of 0.628 ± 0.024 for the multi-PRS approach, while the RR reached only an accuracy of 0.612 ± 0.011. For the single-PRS, the DNN reached an accuracy of 0.613 ± 0.021 and the RR reached an accuracy of 0.598 ± 0.007. For the CAD data set, the DNN reached an accuracy of 0.698 ± 0.012 with the multi-PRS approach, while the RR reached 0.693 ± 0.004. For the single-PRS approach, there were no differences between RR and DNN. Interestingly, using the multi-PRS approach instead of the typically used single-PRS approach generally leads to higher accuracy of the resulting model, irrespective of the underlying prediction model, i.e., RF, RR, or DNN.

## 4 Discussion

We showed that the inclusion of additional PRS improves the prediction quality of PRS models for predicting an individual’s phenotype for BC, CAD, and T2D. The improved prediction quality by including additional PRS can be attributed to the fact that disease susceptibility can be characterized by different risk factors for which at least a partially independent underlying genetic liability exists. For instance, the risk for CAD (coronary artery disease) can be associated with high LDL-cholesterol, high body mass index, smoking, etc., which is also influenced by genetics. Therefore, more comprehensive genetic risk models can be obtained by using a multi-PRS modeling approach. Moreover, by replacing the typically used RR with DNNs, prediction performance could also be improved. DNNs are non-linear classifiers able to capture non-linearity in the underlying data. By not selecting additional PRS manually, we ensured that no information is lost and left it to the algorithms to identify important features. The effect of different PRS on the prediction is likely to be very different. Approaches from explainable AI could be used to identify the relevant PRS.

Although these differences are rather small, the improvement in overall accuracy implies that there are non-linear relationships in the genomics data, as expected from other studies. Improvements in accuracy of up to 1.5%–2% are rather small, but they can have strong implications for patients. For instance, in Europe, there are approximately 355,000 BC cases per year, accounting for more than 90,000 deaths; however, incidences are increasing. Currently, one out of 11 women will develop BC in Europe. In the US, the number is even higher, with approximately 13%, and BC is the second leading cause of death among women. Using prediction models to detect high-risk patients for screening of BC can improve early detection and thus increase life expectancy. An improvement of 1.5% corresponds to more than 5,000 cases that can be detected only in Europe. If we consider T2D, one in 11 adults has diabetes, i.e., 425 million people worldwide. In the United States of America, approximately 11% of people aged between 20 and 79 years have diabetes, while in Europe, it is approximately 6.8%. Approximately 90% of those affected have type 2 diabetes. Every 8 seconds, a person dies as a result of diabetes. It is estimated that almost 700 million people will have diabetes in 2045. Moreover, it has been estimated that a very high number (almost half) of cases are unreported. By improving the risk prediction by 2% solely by incorporating the available data and novel AI models, approximately 7 million more cases could be identified in risk screenings.

From a translational point of view, better prediction performance will improve disease risk stratification. So far, multi-PRS approaches have been rarely applied, mainly due to the limited availability of large-population-based cohorts with deep-phenotyping data to train the model and for the computational issues to deal with high-dimensional data. With the availability of population-based cohorts (such as UK Biobank) and the parallel improvement of computational algorithms for big-data processing, the training of multi-PRS models is feasible on standard HPC infrastructure. Instead, the final application of the models on independent test data is not computationally demanding and therefore can be run locally and potentially integrated into a clinical setting. Additional PRS can be calculated on imputed SNPs based on reference haplotypes if they were not included in the original SNP array.

Our study presents different limitations. In particular, we focused on the genetic predictions of complex traits, including only sex and age as non-genetic factors. However, it is well known that genetic predictors explain only a relatively small proportion of the heritability of complex traits (Gusev et al., 2013). Therefore, in translational settings, different non-genetic risk factors should be included in the prediction models in order to obtain an optimized risk stratification [e.g., the BOADICEA model for breast cancer (Lee et al., 2019)]. Since the multi-PRS model is based on multiple PRS, general limitations of PRS also apply to our model. Some SNPs associated with the diseases may be undiscovered by GWAS, and effect sizes are imprecise (Lewis and Vassos, 2020). Additionally, PRS suffer from a portability problem. PRS calculated on one genetic ancestry perform worse on groups of different ancestry (Martin et al., 2019). In our work, the data set is mainly composed of samples with European genetic backgrounds. Given the different allele frequencies across populations and the limited sample size of non-European individuals, overfitting with respect to the target European population can affect the generalizability of the model. Family-based GWAS are more robust to the effects of population stratification but generally lack power in comparison to non-family-based GWAS (Laird and Lange, 2009). Furthermore, the interpretation of PRS can be difficult and lead to overdiagnosis, resulting in inappropriate treatment (Polygenic Risk Score Task Force of the International Common Disease Alliance et al., 2021).

In the future, we aim to incorporate not only genomics information and PRS but also other clinical data and questionnaires to further improve the risk predictions. As the number of scores in the PGS Catalog constantly grows, those new PRS can be used to update and potentially improve the multi-PRS model. Furthermore, tools other than PLINK (Chang et al., 2015) [e.g., LDpred2 (Privé et al., 2021), PRSice-2 (Choi and O’Reilly, 2019), PRS-CSx (Ruan et al., 2022), or PRSMix (Truong et al., 2023)] can be used to calculate the input PRS.

## Data Availability

Publicly available datasets were analyzed in this study. These data can be found at: UK Biobank.
