# Supplementary material for: AI-based multi-PRS models outperform classical single-PRS models
Source: Front Genet. 2023 Jun 27;14:1217860. doi: 10.3389/fgene.2023.1217860 (PMC10335560; doi:10.3389/fgene.2023.1217860)
Supplement: Supplementary file 1 [file Table1.pdf]

Table 1: Additionally used polygenic risk scores from the Polygenic Score Catalog, their reported trait and number of variants used.

ID	PGS Name	Reported Trait	Number of Variants	citation
PGS000004	PRS313_BC	Breast Cancer	313	Mavaddat N *et al.* *Am J Hum Genet* (2018)
PGS000005	PRS313_ERpos	ER-positive Breast Cancer	313	Mavaddat N *et al.* *Am J Hum Genet* (2018)
PGS000006	PRS313_ERneg	ER-negative Breast Cancer	313	Mavaddat N *et al.* *Am J Hum Genet* (2018)
PGS000007	PRS3820_BC	Breast Cancer	3,820	Mavaddat N *et al.* *Am J Hum Genet* (2018)
PGS000008	PRS3820_ERpos	ER-positive Breast Cancer	3,820	Mavaddat N *et al.* *Am J Hum Genet* (2018)
PGS000009	PRS3820_ERneg	ER-negative Breast Cancer	3,820	Mavaddat N *et al.* *Am J Hum Genet* (2018)
PGS000015	GPS_BC	Breast cancer	5,218	Khera AV *et al.* *Nat Genet* (2018)
PGS000025	GRS	Alzheimer's Disease	19	Chouraki V *et al.* *J Alzheimers Dis* (2016)
PGS000043	PRS_VTE	Venous thromboembolism	297	Klarin D *et al.* *Nat Genet* (2019)
PGS000045	BCPRS_Overall	Breast cancer	88	Kuchenbaecker KB *et al.* *J Natl Cancer Inst* (2017)
PGS000046	BCPRS_ER+	Estrogen receptor [ER]-positive breast cancer	87	Kuchenbaecker KB *et al.* *J Natl Cancer Inst* (2017)
PGS000047	BCPRS_ER-	Estrogen receptor [ER]-negative breast cancer	53	Kuchenbaecker KB *et al.* *J Natl Cancer Inst* (2017)
PGS000048	OCPRS_Overall	Ovarian cancer	17	Kuchenbaecker KB *et al.* *J Natl Cancer Inst* (2017)
PGS000049	PRS103_PrCa	Prostate Cancer	103	Lecarpentier J *et al.* *J Clin Oncol* (2017)
PGS000050	PRS44	Breast cancer	44	Wen W *et al.* *Breast Cancer Res* (2016)
PGS000052	sPRS161	Breast cancer	161	Lakeman IMM *et al.* *J Med Genet* (2019)
PGS000053	ALZ21_NIA-LOAD	Alzheimer's disease (late onset)	21	Tosto G *et al.* *Neurology* (2017)
PGS000054	ALZ21_EFIGA	Alzheimer's disease (late onset)	21	Tosto G *et al.* *Neurology* (2017)
PGS000055	PRS_CRC	Colorectal cancer	76	Schmit SL *et al.* *J Natl Cancer Inst* (2019)

PGS000058	CAD_GRS_204	Coronary artery disease	204	Morieri ML *et al. Diabetes Care* (2018)
PGS000064	GLGC2017_HDL	High density lipoprotein (HDL) cholesterol	120	Kuchenbaecker K *et al. Nat Commun* (2019)
PGS000065	GLGC2017_LDL	Low density lipoprotein (LDL) cholesterol	103	Kuchenbaecker K *et al. Nat Commun* (2019)
PGS000066	GLGC2017_TG	Triglycerides (TG)	101	Kuchenbaecker K *et al. Nat Commun* (2019)
PGS000068	PRS_EOC	Epithelial ovarian cancer	15	Yang X *et al. J Med Genet* (2018)
PGS000069	PRS_sEOC	Serous epithelial ovarian cancer	15	Yang X *et al. J Med Genet* (2018)
PGS000070	PRS_LC_C	Lung cancer	19	Dai J *et al. Lancet Respir Med* (2019)
PGS000071	CC_Bladder	Bladder cancer	15	Graff RE *et al. Nat Commun* (2021)
PGS000072	CC_Breast	Breast cancer	187	Graff RE *et al. Nat Commun* (2021)
PGS000074	CC_Colorectal	Colorectal cancer	103	Graff RE *et al. Nat Commun* (2021)
PGS000075	CC_Endo	Endometrial cancer	9	Graff RE *et al. Nat Commun* (2021)
PGS000076	CC_Kidney	Kidney cancer	19	Graff RE *et al. Nat Commun* (2021)
PGS000077	CC_LL	Lymphocytic leukemia	75	Graff RE *et al. Nat Commun* (2021)
PGS000078	CC_Lung	Lung cancer	109	Graff RE *et al. Nat Commun* (2021)
PGS000079	CC_Melanoma	Melanoma	24	Graff RE *et al. Nat Commun* (2021)
PGS000080	CC_NHL	Non-Hodgkin's lymphoma	19	Graff RE *et al. Nat Commun* (2021)
PGS000081	CC_Oral	Oral cavity and pharyngeal cancers	14	Graff RE *et al. Nat Commun* (2021)
PGS000082	CC_Ovary	Ovarian cancer	36	Graff RE *et al. Nat Commun* (2021)
PGS000083	CC_Pancreas	Pancreatic cancer	22	Graff RE *et al. Nat Commun* (2021)
PGS000084	CC_Prostate	Prostate cancer	161	Graff RE *et al. Nat Commun* (2021)
PGS000085	*retired*	Stomach cancer	-	-
PGS000086	CC_Testis	Testicular cancer	52	Graff RE *et al. Nat Commun* (2021)
PGS000087	CC_Thyroid	Thyroid cancer	12	Graff RE *et al. Nat Commun* (2021)
PGS000088	baso	Basophil count	9,121	Xu Y *et al. Cell Genom* (2022)

PGS000089	baso_p	Basophil percentage of white cells	5,248	Xu Y *et al. Cell Genom* (2022)
PGS000090	eo	Eosinophil count	22,949	Xu Y *et al. Cell Genom* (2022)
PGS000091	eo_p	Eosinophil percentage of white cells	24,406	Xu Y *et al. Cell Genom* (2022)
PGS000092	hct	Hematocrit	28,214	Xu Y *et al. Cell Genom* (2022)
PGS000093	hgb	Hemoglobin concentration	25,090	Xu Y *et al. Cell Genom* (2022)
PGS000094	hlr	High light scatter reticulocyte count	25,493	Xu Y *et al. Cell Genom* (2022)
PGS000095	hlr_p	High light scatter reticulocyte percentage of red cells	21,957	Xu Y *et al. Cell Genom* (2022)
PGS000096	irf	Immature fraction of reticulocytes	17,850	Xu Y *et al. Cell Genom* (2022)
PGS000097	lymph	Lymphocyte count	24,646	Xu Y *et al. Cell Genom* (2022)
PGS000098	lymph_p	Lymphocyte percentage of white cells	22,363	Xu Y *et al. Cell Genom* (2022)
PGS000099	mch	Mean corpuscular hemoglobin	27,081	Xu Y *et al. Cell Genom* (2022)
PGS000100	mchc	Mean corpuscular hemoglobin concentration	11,832	Xu Y *et al. Cell Genom* (2022)
PGS000101	mcv	Mean corpuscular volume	25,001	Xu Y *et al. Cell Genom* (2022)
PGS000102	mono	Monocyte count	28,162	Xu Y *et al. Cell Genom* (2022)
PGS000103	mono_p	Monocyte percentage of white cells	22,843	Xu Y *et al. Cell Genom* (2022)
PGS000104	mpv	Mean platelet volume	25,745	Xu Y *et al. Cell Genom* (2022)
PGS000105	neut	Neutrophil count	23,864	Xu Y *et al. Cell Genom* (2022)
PGS000106	neut_p	Neutrophil percentage of white cells	22,049	Xu Y *et al. Cell Genom* (2022)

PGS000107	pct	Plateletcrit	30,459	Xu Y *et al. Cell Genom* (2022)
PGS000108	pdw	Platelet distribution width	25,995	Xu Y *et al. Cell Genom* (2022)
PGS000109	plt	Platelet count	26,683	Xu Y *et al. Cell Genom* (2022)
PGS000110	rbc	Red blood cell count	23,242	Xu Y *et al. Cell Genom* (2022)
PGS000111	ret	Reticulocyte count	26,077	Xu Y *et al. Cell Genom* (2022)
PGS000112	ret_p	Reticulocyte fraction of red cells	25,939	Xu Y *et al. Cell Genom* (2022)
PGS000113	wbc	White blood cell count	28,383	Xu Y *et al. Cell Genom* (2022)
PGS000115	LDL-C_20	low density lipoprotein cholesterol	223	Trinder M *et al. JAMA Cardiol* (2020)
PGS000124	IOP_AS	Intraocular pressure	103	MacGregor S *et al. Nat Genet* (2018)
PGS000146	CRC_GRS_27	Colorectal cancer risk	27	Hsu L *et al. Gastroenterology* (2015)
PGS000151	SC_GRS	Colorectal cancer	14	Xin J *et al. Gene* (2018)
PGS000163	baso	Basophil count	185	Vuckovic D *et al. Cell* (2020)
PGS000164	baso_p	Basophil percentage of white cells	150	Vuckovic D *et al. Cell* (2020)
PGS000165	eo	Eosinophil count	607	Vuckovic D *et al. Cell* (2020)
PGS000166	eo_p	Eosinophil percentage of white cells	571	Vuckovic D *et al. Cell* (2020)
PGS000167	hct	Hematocrit	502	Vuckovic D *et al. Cell* (2020)
PGS000168	hgb	Hemoglobin concentration	515	Vuckovic D *et al. Cell* (2020)
PGS000169	hlr	High light scatter reticulocyte count	570	Vuckovic D *et al. Cell* (2020)
PGS000170	hlr_p	High light scatter reticulocyte percentage of red cells	566	Vuckovic D *et al. Cell* (2020)
PGS000171	irf	Immature fraction of reticulocytes	372	Vuckovic D *et al. Cell* (2020)
PGS000172	lymph	Lymphocyte count	621	Vuckovic D *et al. Cell* (2020)

PGS000173	lymph_p	Lymphocyte percentage of white cells	472	Vuckovic D *et al. Cell* (2020)
PGS000174	mch	Mean corpuscular hemoglobin	628	Vuckovic D *et al. Cell* (2020)
PGS000175	mchc	Mean corpuscular hemoglobin concentration	224	Vuckovic D *et al. Cell* (2020)
PGS000176	mcv	Mean corpuscular volume	685	Vuckovic D *et al. Cell* (2020)
PGS000177	mono	Monocyte count	638	Vuckovic D *et al. Cell* (2020)
PGS000178	mono_p	Monocyte percentage of white cells	549	Vuckovic D *et al. Cell* (2020)
PGS000179	mpv	Mean platelet volume	654	Vuckovic D *et al. Cell* (2020)
PGS000180	mrv	Mean reticulocyte volume	629	Vuckovic D *et al. Cell* (2020)
PGS000181	mscv	Mean sphered corpuscular volume	761	Vuckovic D *et al. Cell* (2020)
PGS000182	neut	Neutrophil count	492	Vuckovic D *et al. Cell* (2020)
PGS000183	neut_p	Neutrophil percentage of white cells	437	Vuckovic D *et al. Cell* (2020)
PGS000184	pct	Plateletcrit	700	Vuckovic D *et al. Cell* (2020)
PGS000185	pdw	Platelet distribution width	555	Vuckovic D *et al. Cell* (2020)
PGS000186	plt	Platelet count	739	Vuckovic D *et al. Cell* (2020)
PGS000187	rbc	Red blood cell count	678	Vuckovic D *et al. Cell* (2020)
PGS000188	rdw	Red cell distribution width	546	Vuckovic D *et al. Cell* (2020)
PGS000189	ret	Reticulocyte count	555	Vuckovic D *et al. Cell* (2020)
PGS000190	ret_p	Reticulocyte fraction of red cells	537	Vuckovic D *et al. Cell* (2020)
PGS000191	wbc	White blood cell count	636	Vuckovic D *et al. Cell* (2020)
PGS000193	MDD_0.001_Coleman_2020	Major depression	1,138	Coleman JRI *et al. Mol Psychiatry* (2020)
PGS000210	LF279	Lung function (FEV1/FVC)	279	Shrine N *et al. Nat Genet* (2019)

PGS000212	PRS330_LumA	Breast cancer intrinsic-like subtype (luminal A-like)	330	Zhang H *et al. Nat Genet* (2020)
PGS000213	PRS330_LumBHER2neg	Breast cancer intrinsic-like subtype (luminal B/HER2-negative-like)	330	Zhang H *et al. Nat Genet* (2020)
PGS000214	PRS330_LumB	Breast cancer intrinsic-like subtype (luminal B-like)	330	Zhang H *et al. Nat Genet* (2020)
PGS000215	PRS330_HER2	Breast cancer intrinsic-like subtype (HER2-enriched-like)	330	Zhang H *et al. Nat Genet* (2020)
PGS000216	PRS330_TN	Breast cancer intrinsic-like subtype (triple negative)	330	Zhang H *et al. Nat Genet* (2020)
PGS000297	GRS3290_Height	Height	3,290	Xie T *et al. Circ Genom Precis Med* (2020)
PGS000298	GRS941_BMI	Body mass index	941	Xie T *et al. Circ Genom Precis Med* (2020)
PGS000299	GRS462_WHRadjBMI	Waist-to-hip ratio (body mass index adjusted)	462	Xie T *et al. Circ Genom Precis Med* (2020)
PGS000300	GRS80_HR	Heart rate	80	Xie T *et al. Circ Genom Precis Med* (2020)
PGS000301	GRS970_SBP	Systolic blood pressure	970	Xie T *et al. Circ Genom Precis Med* (2020)
PGS000302	GRS962_DBP	Diastolic blood pressure	962	Xie T *et al. Circ Genom Precis Med* (2020)
PGS000303	GRS253_eGFR	Estimated glomerular filtration rate	253	Xie T *et al. Circ Genom Precis Med* (2020)
PGS000304	GRS43_HbA1c	HbA1c	43	Xie T *et al. Circ Genom Precis Med* (2020)
PGS000305	GRS31_FG	Fasting glucose	31	Xie T *et al. Circ Genom Precis Med* (2020)
PGS000306	GRS19_FGadjBMI	Fasting glucose (body mass index adjusted)	19	Xie T *et al. Circ Genom Precis Med* (2020)
PGS000307	GRS12_FI	Fasting insulin	12	Xie T *et al. Circ Genom Precis Med* (2020)
PGS000308	GRS12_FIadjBMI	Fasting insulin (body mass index adjusted)	12	Xie T *et al. Circ Genom Precis Med* (2020)

PGS000309	GRS247_HDL	High-density lipoprotein	247	Xie T *et al. Circ Genom Precis Med* (2020)
PGS000310	GRS194_LDL	Low-density lipoprotein	194	Xie T *et al. Circ Genom Precis Med* (2020)
PGS000311	GRS234_TC	Total cholesterol	234	Xie T *et al. Circ Genom Precis Med* (2020)
PGS000312	GRS190_TG	Triglycerides	190	Xie T *et al. Circ Genom Precis Med* (2020)
PGS000313	GRS49_Lp(a)	Lipoprotein(a)	49	Xie T *et al. Circ Genom Precis Med* (2020)
PGS000314	GRS77_CRP	C-reactive protein	77	Xie T *et al. Circ Genom Precis Med* (2020)
PGS000315	GRS7_lgE	Immunoglobulin E (IgE)	7	Xie T *et al. Circ Genom Precis Med* (2020)
PGS000318	cPRS_F	All-cause mortality (female)	4,122	Meisner A *et al. Am J Hum Genet* (2020)
PGS000319	cPRS_M	All-cause mortality (male)	4,092	Meisner A *et al. Am J Hum Genet* (2020)
PGS000321	snpnet_Testosterone_combined	Serum testosterone levels	7,319	Flynn E *et al. Eur J Hum Genet* (2020)
PGS000322	snpnet_Testosterone_female-specific	Serum testosterone levels in females	7,168	Flynn E *et al. Eur J Hum Genet* (2020)
PGS000323	snpnet_Testosterone_male-specific	Serum testosterone levels in males	8,235	Flynn E *et al. Eur J Hum Genet* (2020)
PGS000324	GRS-JIA-ERA-20	Enthesitis-related Juvenile Idiophatic Arthritis	138	Cánovas R *et al. Ann Rheum Dis* (2020)
PGS000325	GRS-JIA-Oli-20	Oligoarthritis Juvenile Idiophatic Arthritis	21	Cánovas R *et al. Ann Rheum Dis* (2020)
PGS000326	GRS-JIA-RFN-20	Rheumatoid-factor-negative Polyarthritis (Juvenile Idiophatic Arthritis)	12	Cánovas R *et al. Ann Rheum Dis* (2020)
PGS000334	GRSfull_22	Late-onset Alzheimer's disease	22	Zhang Q *et al. Nat Commun* (2020)
PGS000336	GRS313_Chron	Chronotype	313	Maukonen M *et al. J Biol Rhythms* (2020)
PGS000338	GRS97_AF	Atrial fibrillation	97	Kloosterman M *et al. Eur J Heart Fail* (2020)
PGS000339	PRS22_CM	Cutaneous melanoma	22	Law MH *et al. Hum Mol Genet* (2020)
